# Re-Audit of Compliance With Standard Operating Procedure for Prescription of Depot Medication Within the Wolverhampton Older Adult Services

**DOI:** 10.1192/bjo.2022.462

**Published:** 2022-06-20

**Authors:** Clare Siew Boon Ling, Aparna Prasanna

**Affiliations:** 1 New Cross Hospital, Wolverhampton, United Kingdom; 2 Black Country Healthcare NHS Foundation Trust, West Midlands, United Kingdom

## Abstract

**Aims:**

1) To re-audit the current practice of depot prescribing within the Wolverhampton Older Adult Enhanced Community Mental Health Teams (ECMHT). 2) To assess whether the implementation of a memory aid for prescribers has improved compliance of current practice to the Black Country Healthcare Foundation Trust (BCHFT) standard operating procedures (SOP) protocol.

**Methods:**

All depot cards were identified from the Wolverhampton Older Adult ECMHT in January 2022. The cards were assessed for their compliance with the 15 standards for depot prescription writing as set out by the BCHFT SOP protocol. If a standard was not met, reasons for non-compliance were documented. The compliance rate for each standard was then compared to the results from a previous audit performed in January 2021.

**Results:**

A total of 13 depot cards were identified. Out of the 15 standards, 6 of them had a 100% compliance rate. The two standards with the lowest compliance rate were ‘Standard 3’ and ‘Standard 7’. Standard 3 states that “Prescriptions should be signed and dated appropriately, including full signature and name printed”. This standard only achieved 15% compliance. This was a 60% reduction from the previous 75% compliance. Standard 7 states that “The interval expressed should be using the word ‘every’”. This standard achieved a compliance rate of 31%. This was a 12% improvement from the previous 19%.

**Conclusion:**

This re-audit has shown there is still significant room for improvement regarding depot prescribing. The reason for non-compliance to Standard 3 was largely due to prescribers not printing their names alongside their signatures. This is likely due to the lack of an assigned space for “Prescriber's name” to be printed on the form. Also, like the previous audit in 2021, prescribers are still not using the word ‘every’ when filling in the frequency of depots (Standard 7). Despite this, there is a 12% improvement in compliance rate which shows that the memory aid did help some prescribers to comply to Standard 7.

These results will be discussed at the trust's Clinical Audit and Effectiveness Committee meeting. We will then revise the community depot cards to include columns for both prescriber's signature and name. Finally, we will harmonise depot cards from all localities in BCHFT. We will continue to include the memory aid at the front of all depot card folders as it has proven effective. We aim to complete these by March 2022.

